# Subcision—The Art of Controlled Aggression

**DOI:** 10.1111/jocd.16746

**Published:** 2025-01-06

**Authors:** Davin S. Lim

**Affiliations:** ^1^ Cutis Dermatology DVP Woollahra Sydney Australia

To the Editor,

We recently published a manuscript entitled “Complications of Subcision for Acne Scarring: Experience From Clinical Practice and Review of the Literature.” This article describes the various methodologies and instrumentations employed in subcision including the use of hypodermic needles, Nokor needles, cannulas, blunt blades, cataract blades, throught to repurposed dove tail Toledo cannulas and novel sharp‐edged instruments such as the Taylor Liberator.

Different techniques have been described in the literature, ranging from the conservative localized undermining of focal areas, triangulation using longer instruments, and modification of hypodermic needles, through to aggressive field undermining in conjunction with energy devices. There are limited studies on the efficacy and side effects of different techniques due to the polymorphic nature of acne scarring in addition to a lack of standardized execution of the procedure itself. Most studies are multimodality treatments that reflect the variation of scar signatures seen in acne scar patients [1].

Whilst some authors propose restricting the length of instrumentation to minimize collateral damage to retaining ligaments, others recommend employing longer flexible instruments alongside a more conservative approach to mitigate side effects at entry points. Despite initial studies showing positive outcomes with sharp needle instruments, recent research indicates that longer and broader instruments, rather than hypodermic needles, yield better results whilst minimizing operating times [2].

The plane of subcision has been largely agreed upon, namely the hypodermis; however, there is no consensus regarding the extent of undermining of the scar field. With “point subcision” using hypodermic needles, scars are released within a short distance of the chosen instrumentation. In comparison, longer instruments with wide dissecting heads (Taylor Liberators, blunt dissectors, and Toledo cannula) are more efficient at transection of scar tissue, with the drawback of collateral damage to normal tissue between scar areas. Over the past few years, there has been a trend to employ these described instruments as they require significantly less treatment time coupled with superior outcomes [2, 3, 4].

Side effects such as hematomas, fibroplasia, post‐inflammatory hyperpigmentation, entry site scarring, and paresthesia have been documented in the literature [5], however In the past 2 years, I have observed two complications referred from other clinics that have been infrequently reported. Although not solely linked to a single instrument, these issues are more frequently associated with the use of a broad‐based W‐tip tool known as the Taylor Liberator. Most frequently, these side effects have occurred after a single aggressive treatment session.

Firstly, there is the phenomenon of persistent facial swelling lasting (defined by duration greater than 6 months). More frequent cases have been observed in patients who have undergone aggressive, extensive field subcision using the large‐caliber Taylor Liberator. Clinically, this presents as diffuse soft tissue swelling in the lower face and malar areas without a palpable mass. In contrast to fibroplasia, which is characterized by distinct palpation along with specific radiological findings, this type of diffuse facial edema shows no identifiable radiological features [6].

A possible explanation for this phenomenon could be the disruption of the lymphatic network. Patients with severe acne and subsequent scarring have compromised lymphatic structures. Chronic inflammatory conditions, such as rosacea and acne, can predispose patients to Morbihan's disease. The underlying cause is thought to involve impaired lymphatic vessel integrity and drainage due to granulomas, histiocytic infiltrates, and mast cell dysregulation. Performing extensive subcision on individuals with already compromised lymphatic drainage may further compromise lymphatic drainage and compound low‐grade inflammation, potentially exacerbating the edema.

In my experience, persistent swelling responds variably to low‐dose isotretinoin and dilute intralesional corticosteroid. Some cases resolve with prolonged courses of tetracyclines coupled with lymphatic massage.

A second complication I have observed involves reports of increased facial laxity, often accompanied by persistent lower‐face edema. These cases are often secondary to extensive field subcision in the lower face region, again using the implicated instrument.

I believe that laxity is likely to be multifactorial. It can be a consequence of persistent facial swelling as described. Secondly, it can be due to extensive transection in the hypodermal plane (retinacula cutis) in areas over the insertion of the masseteric and mandibular ligaments. In observed cases, patients who most frequently present with this phenomena are middle aged, leading to the possibility of incidental aged related ptosis, which adds to the observation of accelerated sagging of the lower face.

As we strive to better understand the causes of these complications and establish guidelines for the extent of undermining in scar treatment, it may be wise for physicians to adopt a more conservative approach to subcision. It is crucial to respect the underlying structures that maintain facial integrity and recognize that this procedure should be performed with a balanced, controlled technique and controlled aggression.

## Conflicts of Interest

The author declares no conflicts of interest.

## Disclaimer

To date, there is a lack of objective data in the respect of these cases. Reports are anecdotal with a lack of standardized photographic documentation as procedures were performed by other clinicians.

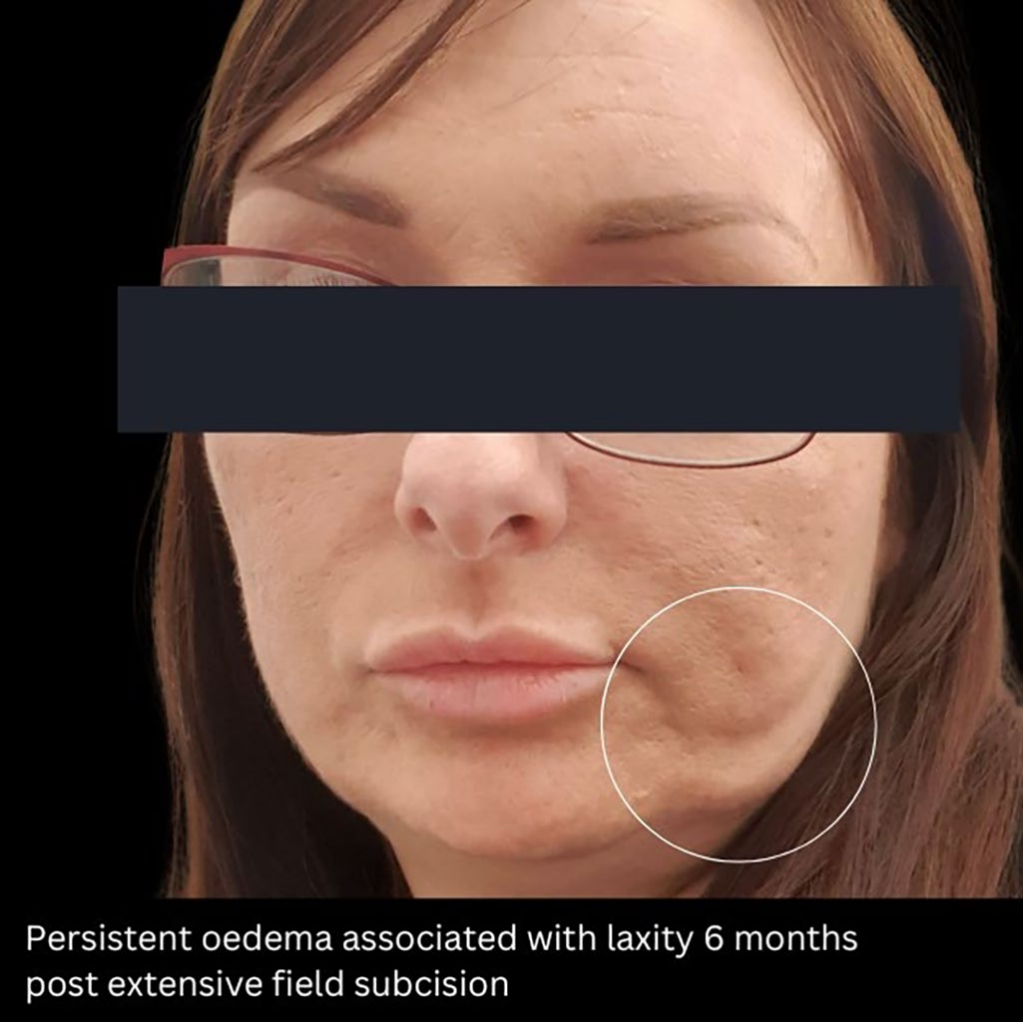

**Figure:**
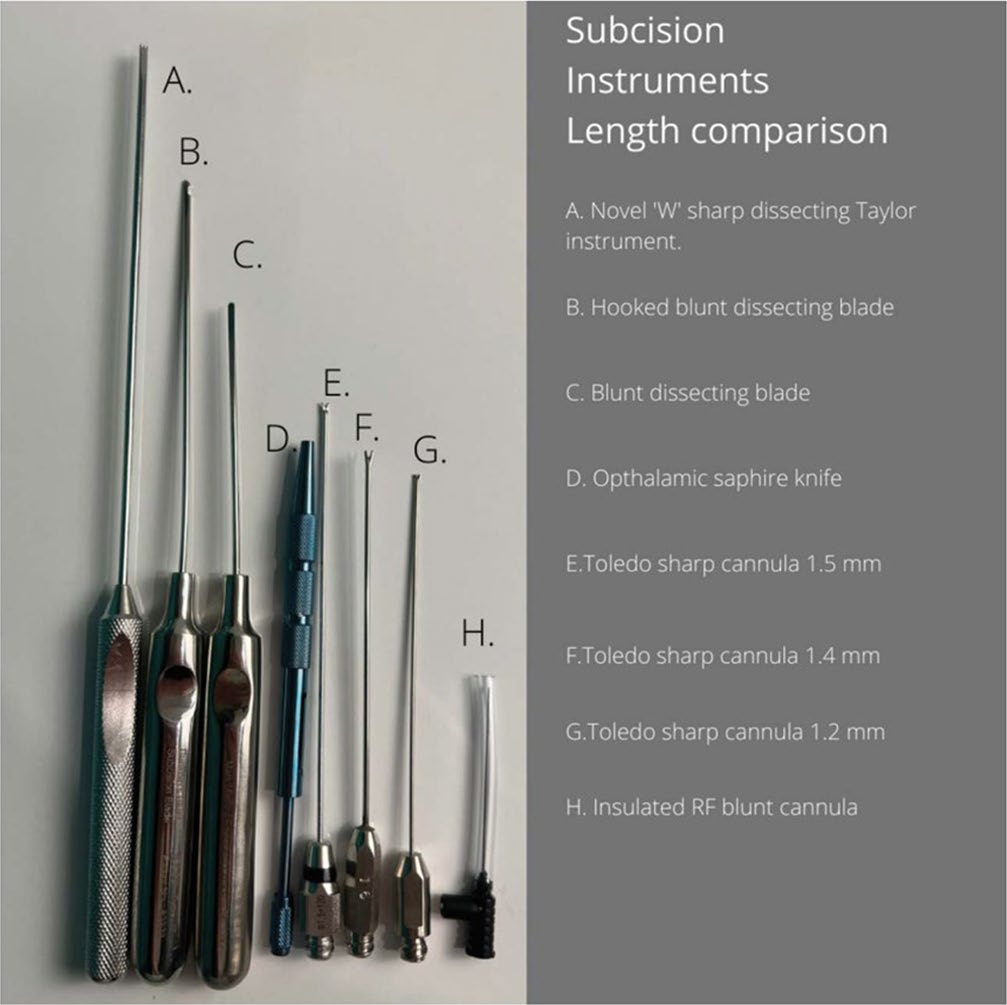
The novel “W” dissection instrument labeled A (Taylor Liberator) has one the broadest instrument heads which lends itself to a highly efficient method of subcision.

## Data Availability

The author has nothing to report.
